# Hsa_circ_0050102 regulates the pancreatic cancer development via miR‐218‐5p/PPME1 axis

**DOI:** 10.1002/jcla.24247

**Published:** 2022-01-21

**Authors:** Ningning Feng, Zhikai Jiao, Yueshan Zhang, Baoming Yang

**Affiliations:** ^1^ Department of Hepatobiliary Surgery Fourth Hospital of Hebei Medical University Shijiazhuang China

**Keywords:** hsa_circ_0050102 migration, miR‐218‐5p, pancreatic cancer, PPME1

## Abstract

**Background:**

Pancreatic cancer (PC) is a malignancy worldwide. Circular RNAs (circRNAs) affects the growth of PC, nonetheless the mechanism is blurry. Here, we reconnoitered the parts of hsa_circ_0050102 in PC.

**Methods:**

Hsa_circ_0050102, microRNA‐218‐5p (miR‐218‐5p) and protein phosphatase methylesterase 1 (PPME1) abundances were indicated by quantitative RT‐PCR or Western blot. Moreover, the cell functions were uncovered. Additionally, the relation of miR‐218‐5p and hsa_circ_0050102 or PPME1 was identified by dual‐luciferase reporter assay. Ultimately, the mice teats were utilized to quantity the part of hsa_circ_0050102.

**Results:**

Hsa_circ_0050102 and PPME1 contents were increased, and the miR‐218‐5p was dwindled in PC. Hsa_circ_0050102 lack subdued cell vitality, colony formation, cell migration and invasion, and angiogenesis, but endorsed cell apoptosis in PC cells. Furthermore, miR‐218‐5p was established to block the development of PC cells via PPME1. Hsa_circ_0050102 bound to miR‐218‐5p to adjust the content of PPME1.

**Conclusion:**

Hsa_circ_0050102 expedited the expansion of PC through growing PPME1 abundance by adjusting miR‐218‐5p.

## Graphical abstract

Circ_0050102 promoted proliferation, migration and invasion, and angiogenesis, while repressed cell apoptosis of PC cells via miR‐218‐5p/PPME1 axis.

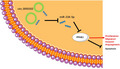

## 1 INTRODUCTION

Pancreatic cancer (PC) is a common cancer with great morbidity and mortality.^1^ The incidence of PC is closely related to smoking, genetics, diabetes, obesity, diet, age and other factors.^2^, ^3^, ^4^, ^5^ Besides, the occurrence of PC rises with age, with almost 90% of cases happening in people over 55.^6^, ^7^ Therefore, we urgently need to find a new way to treat and diagnose PC.

Circular RNAs (circRNAs) are RNAs that play vital special effects in lots of diseases.^8^, ^9^ For instance, hsa_circ_0050102 had stimulative effects in PC.^10^ Besides, circ‐LDLRAD3 was a marker in the judgement of PC.^11^ CircNFIB1 inhibited lymphatic metastasis in PC.^12^ In addition, circPDE8A promoted invasive growth in PC.^13^ Circ_000864 could repress the migration and invasion in PC.^14^ But, the regulatory mode of hsa_circ_0050102 on PC is not clear.

MicroRNAs (miRNAs) have been described in a lot of diseases.^15^, ^16^ For example, miR‐218‐5p regulated the progress of PC.^17^ Besides, miR‐218‐5p regulated the sensitivity to gemcitabine in gallbladder cancer.^18^ Moreover, miR‐218‐5p acted as a marker in PC.^19^ Moreover, miR‐218‐5p took part in adjusting osteosarcoma cancer advancement.^20^ However, the effect of miR‐218‐5p in PC is indistinct.

Protein phosphatase methylesterase 1 (PPME1) is a PP2A‐specific methylesterase, which is an important regulator of tumorigenesis.^21^ For instance, PPME1 acted as an innovative healing target in gastric and lung cancer.^22^ Besides, PPME1 regulated the progression of PC.^23^ In addition, PPME1 promoted choriocarcinoma cell invasion.^24^ However, the behaviour of PPME1 in PC is still blurry.

Here, we revealed the utility of hsa_circ_0050102 in PC cells. Hsa_circ_0050102 might expedite PC by binging miR‐218‐5p and growing the PPME1 content. Our conclusions may be an innovative perception for PC.

## 2 MATERIALS AND METHODS

### 2.1 Clinical tissue samples

The research was appraised by Fourth Hospital of Hebei Medical University. All tissues were gathered from Fourth Hospital of Hebei Medical University, including 60 pairs of PC tissue and normal tissue, tissues of 23 patients with stage I‐II PC, tissues of 37 patients with stage III PC, tissues of 20 patients with LN(–) PC and tissues of 40 patients with LN(+) PC. All the participants offered the written informed consent.

### 2.2 Cell lines

The human PC cell lines (SW1990 and Capan‐1) with HPDE as control. HUVEC cells were selected for the tube formation assay. All cells were gathered from Cell Bank, Chinese Academy of Sciences (CAS, Shanghai, China) and cultured with 5% CO_2_.

### 2.3 Quantitative RT‐PCR and RNase R assay

The tangible method of RNA abstraction and qRT‐PCR were performed conforming to the erstwhile commentary.^25^ The glyceraldehyde‐3‐phosphate dehydrogenase (GAPDH) and U6 were engaged as controls. The gene contents were estimated by 2^−ΔΔCt^ mode. As well, the RNase R (Solarbio) was enforced to estimate the structure of hsa_circ_0050102. The primers as Table 1.

**TABLE 1:** Primers for qRT‐PCR

Name		Primers for PCR (5′‐3′)
hsa_circ_0050102	Forward	CCAGAGAGCGCATTATCCCC
	Reverse	TTTGCCAAGACCCACCTGAT
PPME1	Forward	TTGCCCTCGGAGACTCAGAA
	Reverse	CTACCTTGTCAGGGGCATCC
miR‐218‐5p	Forward	GCCGAGTTGTGCTTGATCTAACC
	Reverse	CTCAACTGGTGTCGTGGA
GAPDH	Forward	TCCCATCACCATCTTCCAGG
	Reverse	GATGACCCTTTTGGCTCCC
U6	Forward	CTCGCTTCGGCAGCACATATACT
	Reverse	ACGCTTCACGAATTTGCGTGT
PGPEP1	Forward	AGGAAGGCGGTGGTAGTGA
	Reverse	GAGATATCTGTGGACTGTGCTT

### 2.4 Western blot

The concrete technique of western blot was corresponding to the prior article.^26^ The antibodies as listed: anti‐PPME1 (clone 4A12; 1:2000; GenWay Biothech, 20‐614‐460633), anti‐PCNA (ab92552; 1:1,000; Abcam), anti‐Bax (ab32503; 1:1,000; Abcam), anti‐Bcl‐2 (ab32124; 1:500; Abcam) and anti‐β‐actin (ab8227; 1:1000; Abcam).

### 2.5 Cell transfection

The si‐hsa_circ_0050102, sh‐hsa_circ_0050102, the si‐NC and sh‐NC, miR‐218‐5p mimics, inhibitors and controls, PPME1 and pcDNA were attained from Ribobio. Transfection was executed with Lipofectamine 2000 (Solarbio).

### 2.6 Cell Counting Kit‐8 (CCK8) assay

After post‐transfection, SW1990 and Capan‐1 (2.0 × 10^3^/well) cells were sowed in 96‐well plates. CCK8 (Solarbio) was supplemented. The absorbance at 450 nm was measured.

### 2.7 Colony formation assay

SW1990 and Capan‐1 (1 × 10^6^) cells were plated in 6‐well plates and then preserved for 10 days. Then, colony was stained and photographed under a microscope.

### 2.8 Transwell assay

SW1990 and Capan‐1 cells were assessed by a transwell with an 8 μm pore polycarbonate membrane (BD Bio‐sciences, Bedford). In brief, 4 × 10^5^ transfected SW1990 and Capan‐1 cells, resuspended in 100 µl of DMEM without serum, were planted into the top chamber. Then the lower chamber of the transwell containing 500 µl of DMEM and 10% FBS. Following this, the moved cells were stained. The same method was enforced to detect the invasion ability, but the chamber with matrigel (BD Biosciences). Eventually, a light microscope was performed to validate the count of cells.

### 2.9 Matrigel tube formation assay

HUVEC cells were implanted into 96‐well plat at 4 × 10^4^ cells/well in Matrigel‐coated wells. Meanwhile, the tube formation rate was examined after 24 h. Afterwards, ImageJ software (NIH, Bethesda) was utilized to observe the number of tubes and the count of branches. The elongated multi‐cellular structures were considered tube‐like structures. The intersecting points of two or more tubes were considered branches.

### 2.10 Flow cytometry assay

The 2 × 10^4^ SW1990 and Capan‐1 cells were planted into 6‐well plates. Then, an Annexin V‐FITC/PI kit (Solarbio) was engaged with a Flow cytometer (BD Biosciences).

### 2.11 Dual‐luciferase reporter assay

The binding sites between miR‐218‐5p and hsa_circ_0050102 or PPME1 were foretold by starbase and targetscan. At that time, the hsa_circ_0050102 and PPME1 wild and mutant were produced by Ribobio (WT‐hsa_circ_0050102, WT‐PPME1 3’UTR or MUT‐hsa_circ_0050102, MUT‐PPME1 3’UTR). Finally, the luciferase activity was scrutinized.

### 2.12 RNA Immunoprecipitation (RIP) assay

SW1990 and Capan‐1 were engaged with a RIP kit (Solarbio) in accordance with the guide to implement RIP assay. Succeeding, the abundances of miR‐218‐5p, hsa_circ_0050102 and PPME1 were illustrated.

### 2.13 Xenograft models

The test was noticed by the Animal Care and Use Committee of Fourth Hospital of Hebei Medical University. All mice were acquired from Shanghai Laboratory Animal Company (SLAC). Capan‐1 cells (5 × 10^6^) with sh‐hsa_circ_0050102 or sh‐NC were vaccinated into mice (*n =* 6/group; female; 6 weeks; 18–22 g). To end, tumour volume = length × width^2^ × 0.5. After 4 weeks, the tumours were carved for extra test.

### 2.14 IHC assay

The Ki67 (ab92742; 1:1,000; Abcam) abundance in tumour were evaluated by IHC assay. The exhaustive mode is in the company of the explanation of Ma et al.^27^ Eventually, the tumour slides were photographed.

### 2.15 Statistical assay

The statistics were collected from no less than 3 recurrences. Student's *t* test and ANOVA were employed in SPSS 17.0 to evaluate the difference. *p* < 0.05 was significant.

## 3 RESULTS

### 3.1 Hsa_circ_0050102 was enhanced in PC

**FIGURE 1:**
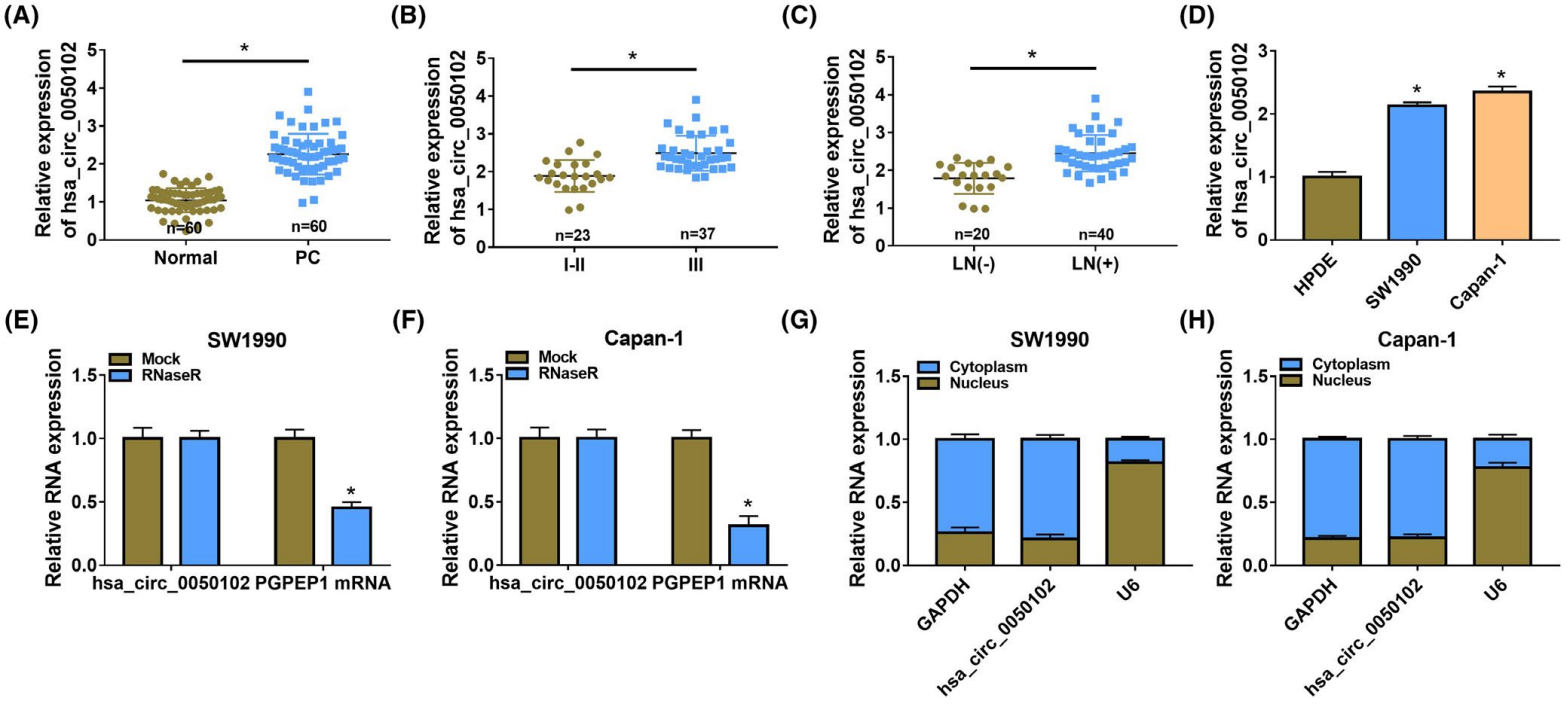
Hsa_circ_0050102 was boosted in PC. (A and B) The abundance of hsa_circ_0050102 was detected by qRT‐PCR. (C) The content of hsa_circ_0050102 in LN (–) PC tissues (*n =* 20) and LN (+) PC tissues (*n =* 40) was distinguished by qRT‐PCR. (D) The hsa_circ_0050102 level in SW1990, Capan‐1 and HPDE cells was unfolded by qRT‐PCR. (E and F) The relative levels of hsa_circ_0050102 and PGPEP1 mRNA were determined. (G and H) The hsa_circ_0050102 content was measured. *p < 0.05

The abundance of hsa_circ_0050102 in PC tissues was augmented (Figure 1A). And, high hsa_circ_0050102 was frequently observed in PC patients with the advanced clinical stage (Figure 1B) and lymph node metastasis (Figure 1C). Additionally, hsa_circ_0050102 was impelled in PC cell lines (SW1990 and Capan‐1) versus HPDE cells (Figure 1D). As exposed in Figure 1E,F, the abundance of PGPEP1 mRNA was significantly abridged after RNase R treatment, but hsa_circ_0050102 was not altered. The consequence illustrated the cyclical construction of hsa_circ_0050102. Furthermore, hsa_circ_0050102 was mostly disseminated in the cytoplasm relative to the nuclear (Figure 1G,H).

### 3.2 Hsa_circ_0050102 lack promoted cell apoptosis, while restrained cell proliferation, cell migration and invasion and angiogenesis in PC cells

**FIGURE 2:**
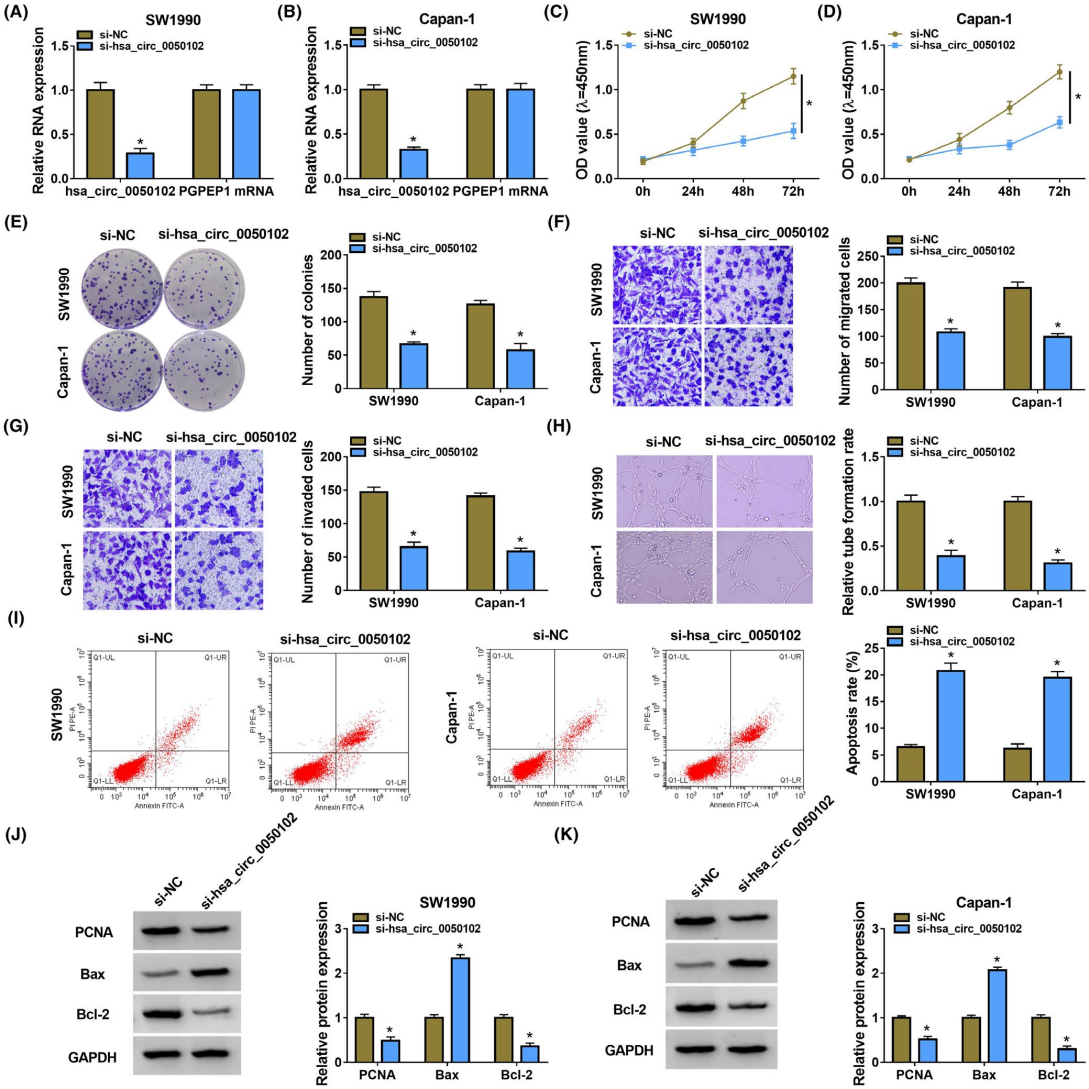
Hsa_circ_0050102 lack subdued PC. (A and B) The content of hsa_circ_0050102 was quantified. (C–E) The cell proliferation was detected. (F and G) The cell migration and invasion were assessed. (H) The tube formation assay assessed the rate of tube formation. (I) The apoptosis was detected. (J and K) The PCNA, Bcl‐2 and Bax contents were examined. *p < 0.05

The hsa_circ_0050102 content was delimited in SW1990 and Capan‐1 cells by si‐hsa_circ_0050102 (Figure 2A,B). The hsa_circ_0050102 lack diminished the cell proliferation (Figure 2C–E). Furthermore, the silence of hsa_circ_0050102 subdued the cell migration and invasion of SW1990 and Capan‐1 cells (Figure 2F,G). Next, the tube formation assay unfolded that silence of hsa_circ_0050102 diminished the cells angiogenesis ability in contrast to controls (Figure 2H). Moreover, hsa_circ_0050102 absence encouraged cell apoptosis in SW1990 and Capan‐1 cells (Figure 2I). PCNA, Bax and Bcl‐2 were demonstrated to be linked with cell proliferation or apoptosis of PC cells. At this time, we verified that si‐hsa_circ_0050102 augmented the abundance of Bax, but abridged the PCNA and Bcl‐2 contents (Figure 2J,K). Our consequences signposted that hsa_circ_0050102 absence might promote cell apoptosis, whereas inhibiting cell vitality, colony formation, cell migration and invasion, and cell tube formation in PC cells.

### 3.3 MiR‐218‐5p targeted hsa_circ_0050102

**FIGURE 3:**
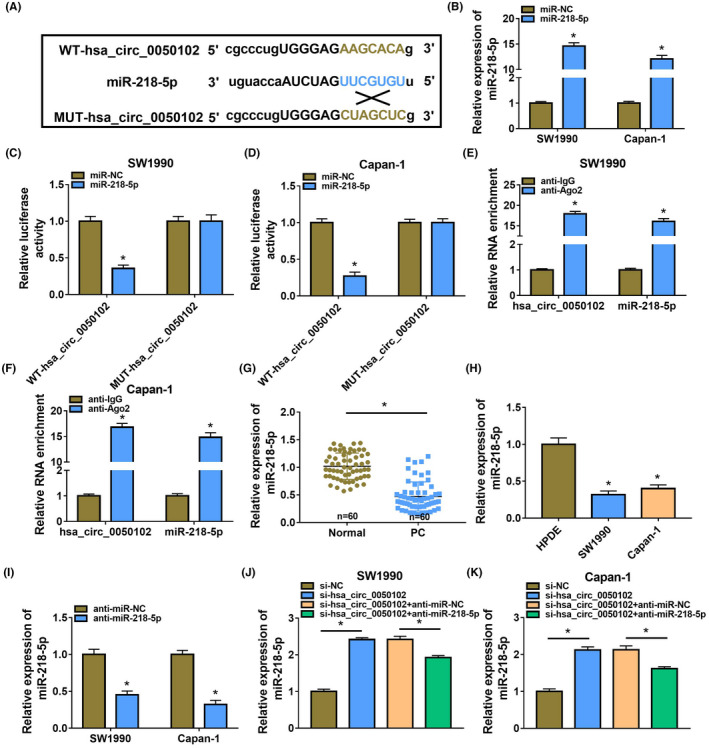
Hsa_circ_0050102 sponged miR‐218‐5p. (A) The bound miRNAs of hsa_circ_0050102 were prediction. (B) The content of miR‐218‐5p in PC cells was detected. (C–F) The relationship of hsa_circ_0050102 and miR‐218‐5p was assessed. (G) The abundance of miR‐218‐5p in PC tissues was detected. (H) The miR‐218‐5p content in PC cells was quantified. (I–K) The miR‐218‐5p content was distinguished. *p < 0.05

**FIGURE 4:**
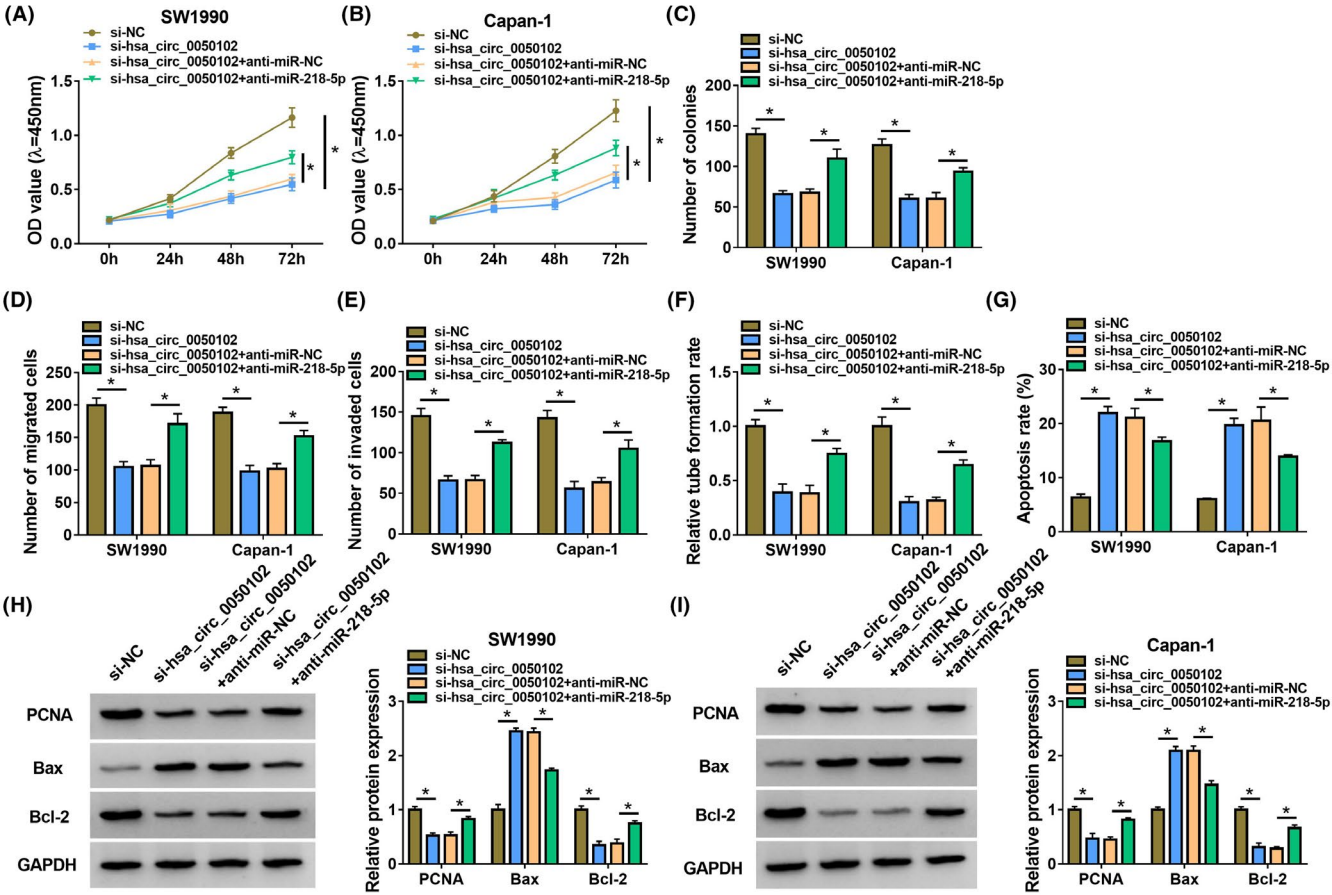
Hsa_circ_0050102 expedited PC via miR‐218‐5p. (A–C) The cell proliferation, (D and E) the cell migration and invasion. (F) the rate of tube formation, (G) the apoptosis, (H and I) the PCNA, Bcl‐2 and Bax levels were quantified. *p < 0.05

MiR‐218‐5p targeted hsa_circ_0050102 (Figure 3A). After miR‐218‐5p mimic treatment, the miR‐218‐5p level augmented (Figure 3B). Meanwhile, the luciferase activity was diminished in WT‐hsa_circ_0050102 with miR‐218‐5p in SW1990 and Capan‐1 cells, but there no alteration in the MUT‐hsa_circ_0050102 group (Figure 3C,D). The RIP assays further authenticated the mutuality of miR‐218‐5p and hsa_circ_0050102 in SW1990 and Capan‐1 cells (Figure 3E,F). Additionally, miR‐218‐5p content was abridged in PC (Figure 3G). In addition, miR‐218‐5p content was dwindled in SW1990 and Capan‐1 cells compared with that in HPDE cells (Figure 3H). Moreover, miR‐218‐5p abundance was declined by miR‐218‐5p lack, whereas it was increased by si‐hsa_circ_0050102 in SW1990 and Capan‐1 cells (Figure 4I–K).

### 3.4 Hsa_circ_0050102 expedited PC via miR‐218‐5p

Initially, hsa_circ_0050102 lack lessened the cell proliferation, but this upshot was abridged by miR‐218‐5p absence (Figure 4A–C). Moreover, silence of hsa_circ_0050102 reduced the cell migration and invasion, yet this influence was diminished by anti‐miR‐218‐5p (Figure 4D,E). In addition, the silence of hsa_circ_0050102 declined the cell's angiogenesis ability; however, this upshot was lessened by anti‐miR‐218‐5p (Figure 4F). Moreover, hsa_circ_0050102 lack prompted cell apoptosis, nevertheless this conclusion was diminished by anti‐miR‐218‐5p (Figure 4G). Furthermore, anti‐miR‐218‐5p reserved the influences of hsa_circ_0050102 lack on augmented the abundance of Bax and decreased the contents of PCNA and Bcl‐2 in SW1990 and Capan‐1 cells (Figure 4H,I).

### 3.5 MiR‐218‐5p targeted PPME1 in PC cells

**FIGURE 5:**
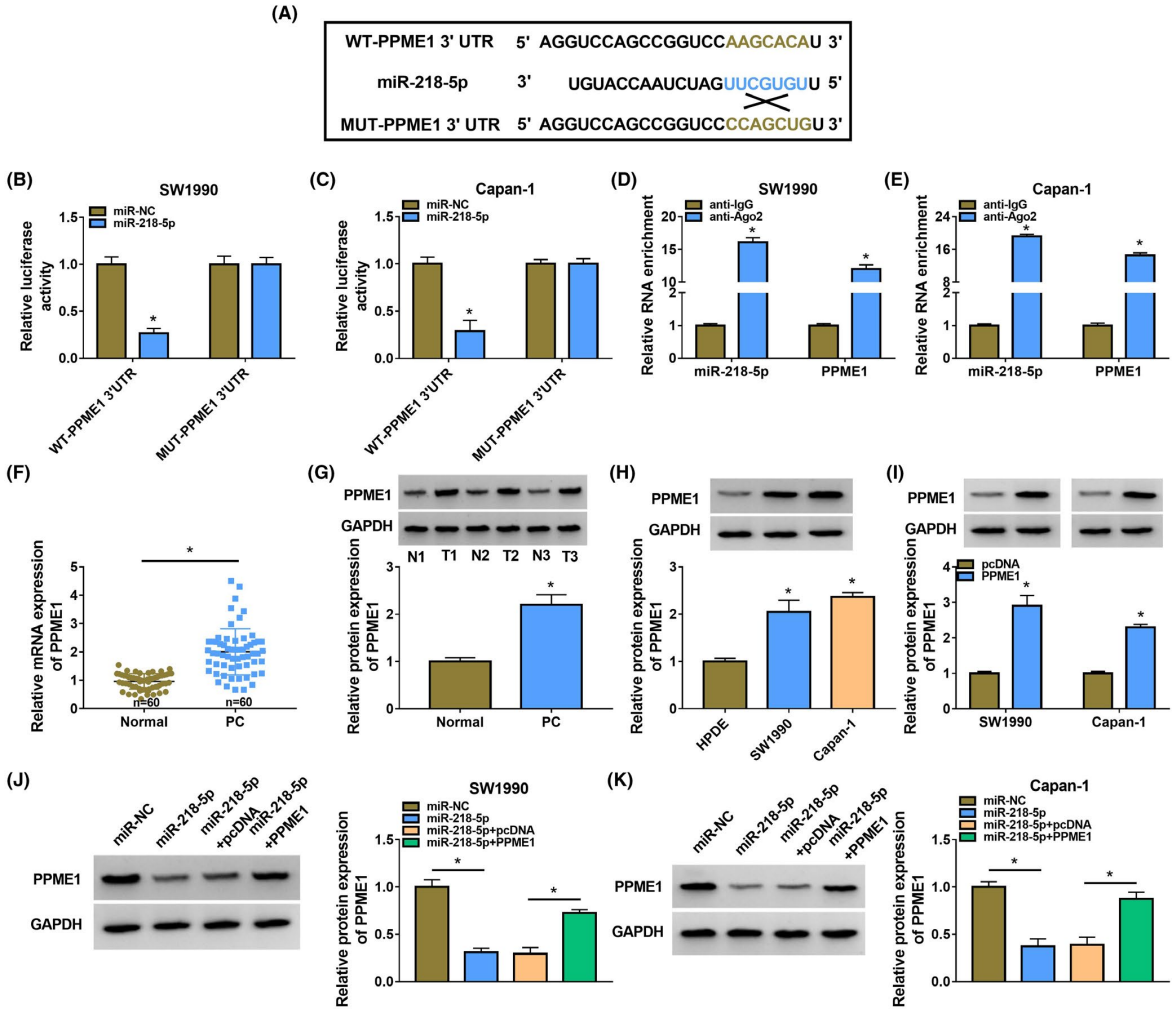
MiR‐218‐5p targeted PPME1 in PC cells. (A) The bound site between miR‐218‐5p and PPME1 was investigated by targetscan. (B–E) The relationship of miR‐218‐5p and PPME1 was evaluated. (F and G) The PPME1 content was detected. (H–K) The level of PPME1 in PC cells was distinguished. *p < 0.05

The targeted sites of miR‐218‐5p in PPME1 3’UTR (Figure 5A). The luciferase activity of WT‐PPME1 3’UTR was dwindled by miR‐218‐5p mimic, but in the MUT‐PPME1 3’UTR group, it was not altered (Figure 5B,C). RIP assay revealed the relation of miR‐218‐5p and PPME1 in SW1990 and Capan‐1 cells (Figure 5D,E). The PPME1 levels were boosted in PC tumour tissues (Figure 5F,G). Furthermore, the abundance of PPME1 was higher in SW1990 and Capan‐1 cells versus HPDE (Figure 5H). Further, the PPME1 level was enlarged by PPME1 versus the pcDNA group in SW1990 and Capan‐1 cells (Figure 5I). Figure 5J,K displayed that the PPME1 content was diminished by miR‐218‐5p and amplified by PPME1 overexpression.

### 3.6 MiR‐218‐5p blocked PC via PPME1

**FIGURE 6:**
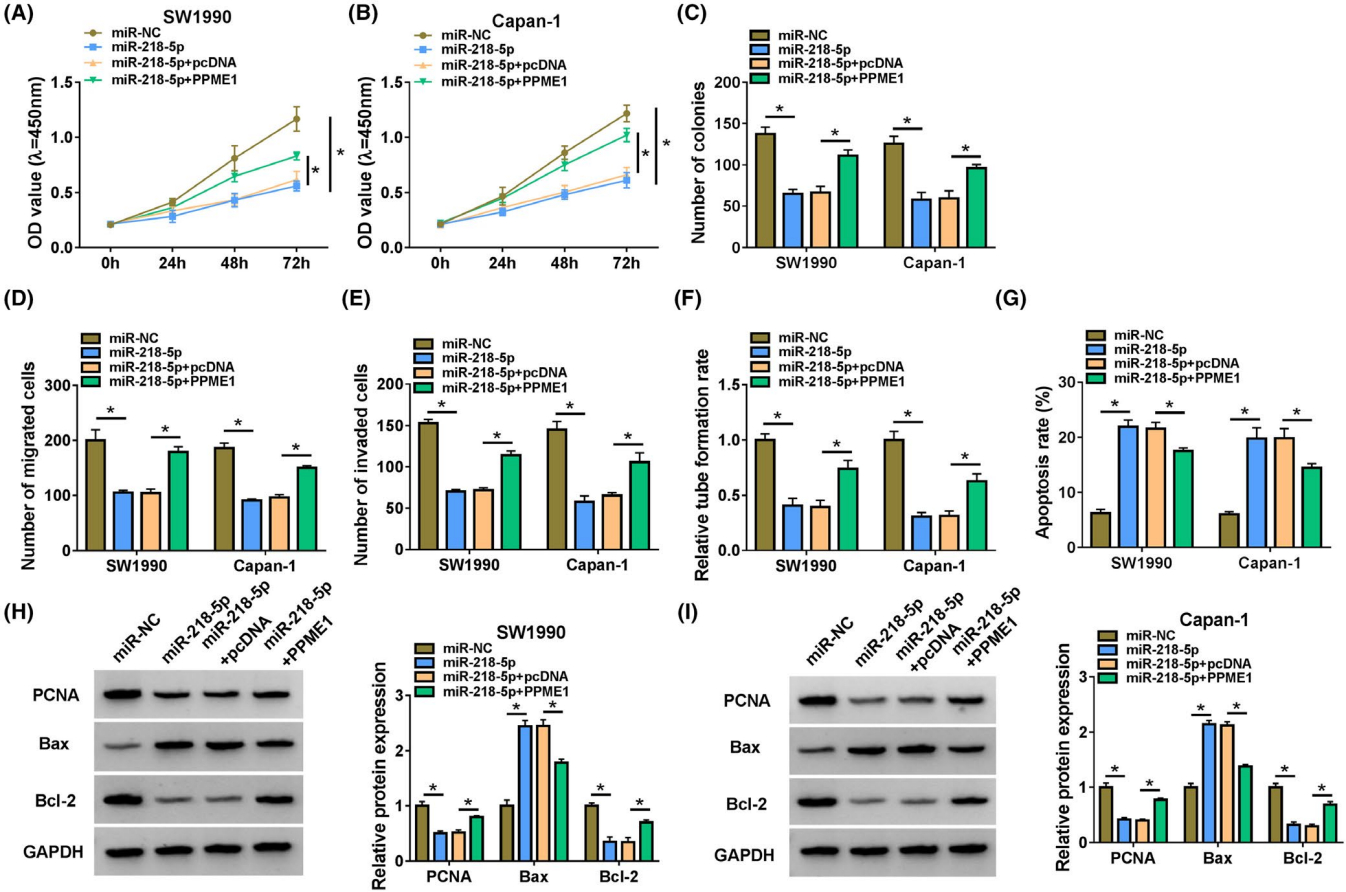
MiR‐218‐5p controlled PC via PPME1. (A–C) The cell proliferation, (D and E) the cell migration and invasion, (F) the rate of tube formation, (G) the apoptosis, (H and I) the PCNA, Bcl‐2 and Bax contents were scrutinized. *p < 0.05

**FIGURE 7:**
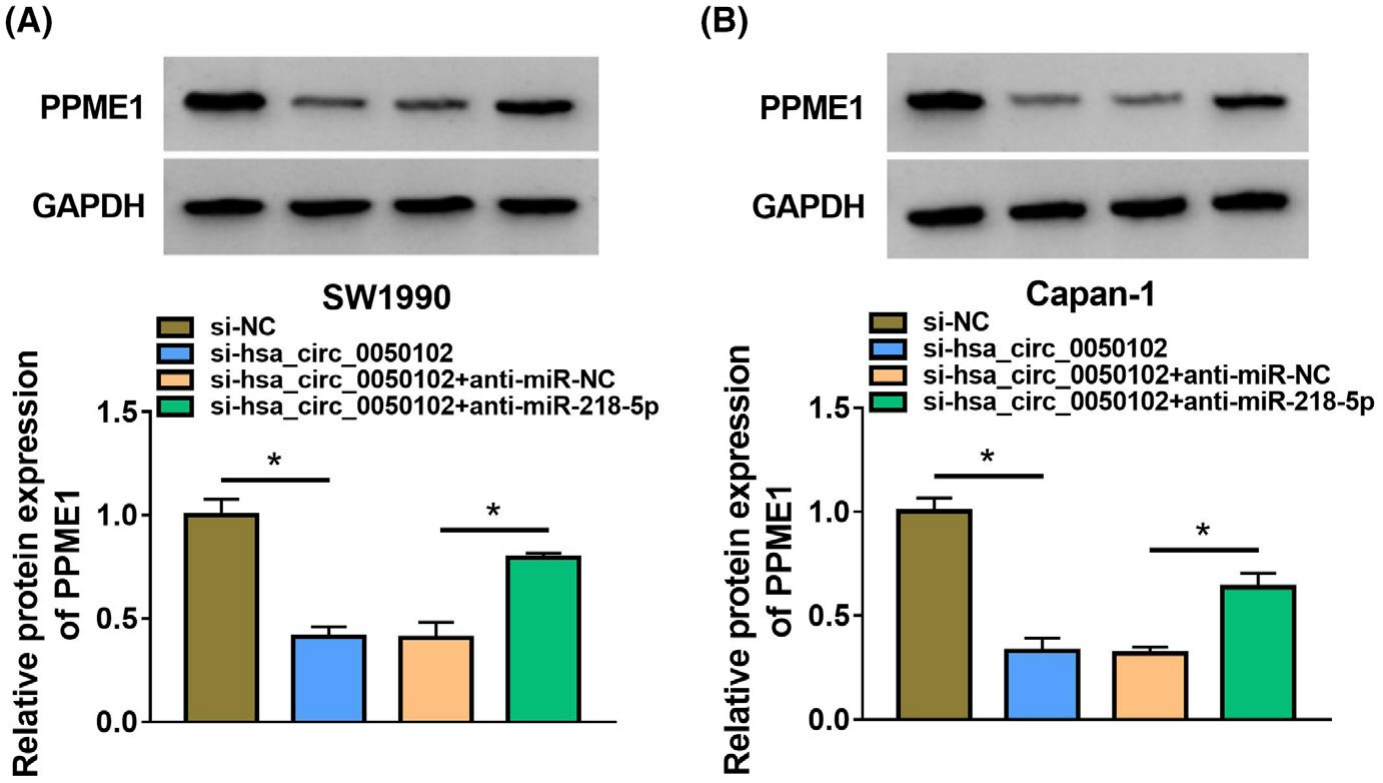
The abundance of PPME1 was adjusted by hsa_circ_0050102 and miR‐218‐5p. (A and B) The level of PPME1 was identified. *p < 0.05

Initially, the miR‐218‐5p enhanced the reserved cell proliferation; nevertheless, this impression was weakened by PPME1 (Figure 6A–C). Besides, miR‐218‐5p blocked the cell migration and invasion, nonetheless this influence was weakened by PPME1 (Figure 6D,E). Besides, miR‐218‐5p curbed the cell angiogenesis viability, but this influence was neutralized by PPME1 (Figure 6F). Afterwards, the miR‐218‐5p expedited cell apoptosis in SW1990 and Capan‐1 cells, and this outcome was alleviated by PPME1 (Figure 6G). The Bax content was augmented and PCNA, Bcl‐2 was lessened by miR‐218‐5p mimic, while PPME1 minimized these effects (Figure 6H,I). In the meantime, the abundance of PPME1 was moderated by si‐hsa_circ_0050102, but anti‐miR‐218‐5p declined the consequences (Figure 7A,B).

### 3.7 Hsa_circ_0050102 lack hampered tumour growth in vivo

**FIGURE 8:**
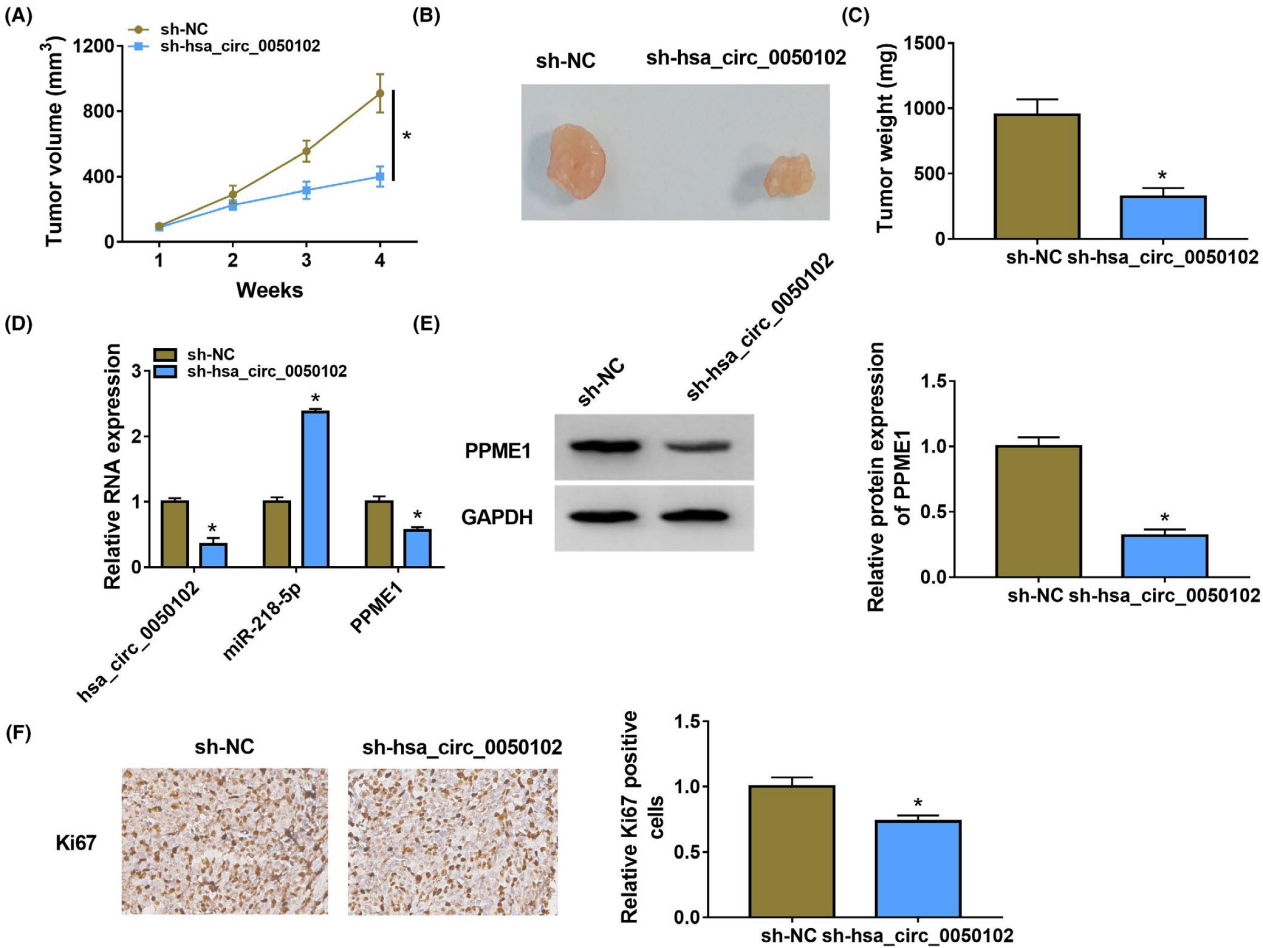
Hsa_circ_0050102 lack curbed tumour growth. (A–C) Tumour growth and weight were measured. (D) The hsa_circ_0050102, miR‐218‐5p and PPME1 contents were identified. (E) The PPME1 level was evaluated. (F) The Ki‐67 level was quantified. *p < 0.05

As revealed in Figure 8A–C, sh‐hsa_circ_0050102 dramatically inhibited tumour volume and weight. Additionally, the abundances of hsa_circ_0050102 and PPME1 were abridged, while the miR‐218‐5p content was enlarged in the sh‐hsa_circ_0050102 group (Figure 8D,E). The content of Ki67 was lesser in the sh‐hsa_circ_0050102 group, which signposted that hsa_circ_0050102’s lack subdued tumour growth (Figure 8F). These upshots approved that hsa_circ_0050102 lack repressed xenograft tumour growth via miR‐218‐5p/PPME1 axis in vivo.

## 4 DISCUSSION

Pancreatic cancer has a very high mortality rate and a very poor prognosis.^1^ There is no valid screening method for PC, so primary prevention of the disease is crucial. The best prevention strategy for PC is to maintain a healthy lifestyle, such as smoking cessation, weight control and exercise.^2^, ^7^, ^28^, ^29^ Nevertheless, the character of circRNA in PC is uncertain. Hence, our article scrutinized the character of hsa_circ_0050102.

Some circRNAs are vital for PC. Such as, circ_001587 inhibited angiogenesis of PC cells.^30^ Moreover, circFOXK2 regulated the advancement of PC.^31^ In our article, we signposted that silence of hsa_circ_0050102 induced cell apoptosis, while inhibiting the evolution of PC cells. CircRNAs could influence gene and emulatively sponged miRNAs, like circ_001587 targeted miR‐223 and circFOXK2 regulated miR‐942 in PC.^30^, ^31^ In this article, hsa_circ_0050102 accelerated PC development by binding miR‐218‐5p. MiR‐218‐5p could adjust the advancement of breast cancer, retinoblastoma and gastric cancer.^32^, ^33^, ^34^ Here, we established the suppression role of miR‐218‐5p in cell advance via PPME1. The upshots certified that PPME1 might take part in the growth of PC.

At present, numerous papers have shown that PPME1 played an exact central role in choriocarcinoma cell migration and invasion by endopeptidase catalytic subunit IMP1 regulation.^23^ Herein, the PPME1 level was upregulated in PC. The miR‐218‐5p subdued cell growth and this impression was declined by PPME1. The anti‐miR‐218‐5p curbed the confined upshot of hsa_circ_0050102’s lack on PPME1 content in PC cells. These outcomes backup the monitoring character of the hsa_circ_0050102/miR‐218‐5p/PPME1 in PC cells.

In brief, hsa_circ_0050102 and PPME1 were upregulated and miR‐218‐5p was downregulated in PC. Additionally, our article firstly established that hsa_circ_0050102 lack promoted cell apoptosis, but blocked PC cells growth via miR‐218‐5p/PPME1 axis. This mechanism might be extra verified by clinical tests someday. We had faith in that this data provided a new mode for the improvement of PC.

## CONFLICT OF INTEREST

The authors declare that they have no conflicts of interest.

## Data Availability

The datasets used and analysed during this study are available from the corresponding author on reasonable request.
